# Tensile Modulus of Polymer Halloysite Nanotube Systems Containing Filler–Interphase Networks for Biomedical Requests

**DOI:** 10.3390/ma15134715

**Published:** 2022-07-05

**Authors:** Yasser Zare, Kyong Yop Rhee, Soo-Jin Park

**Affiliations:** 1 Biomaterials and Tissue Engineering Research Group, Department of Interdisciplinary Technologies, Breast Cancer Research Center, Motamed Cancer Institute, ACECR, Tehran 1125342432, Iran; y.zare@aut.ac.ir; 2 Department of Mechanical Engineering (BK21 Four), College of Engineering, Kyung Hee University, Yongin 449-701, Korea; 3 Department of Chemistry, Inha University, Incheon 22212, Korea

**Keywords:** halloysite nanotube (HNT), nanocomposite, modulus, interphase zone, network

## Abstract

To date, there have been a limited number of studies modeling the tensile modulus in the polymer halloysite nanotube (HNT) systems before or after percolation onset. In this paper, an equation for a composite’s modulus post-percolation onset was developed for HNT-filled samples including the interphase and HNT network. The dispersed nanoparticles and adjoining interphase part were neglected, because they caused ineffective influences on the stiffness of the system after percolation onset. The developed model reflects the impacts of HNTs’ size, interphase depth, percolation onset and the volume shares and moduli of the HNT network and its adjacent interphase on the modulus of HNT-based systems. The impacts of issues on the nanocomposite modulus are defendable, confirming the effectiveness of the developed model. HNT length, interphase depth, HNT concentration, net modulus and net portion directly influenced the stiffness, while the HNT radius and percolation onset had inverse effects. Results show that there was a 142% improvement in the modulus of samples at an interphase depth of 40 nm. Moreover, the stiffness improved by 60% at a net modulus of 200 GPa, but it later exhibited a 180% enhancement at a net modulus of 1000 GPa. In addition, the experimental data for the modulus of numerous composites display fine agreement to the predictions, confirming the validity of the developed model.

## 1. Introduction

**Figure 1:**
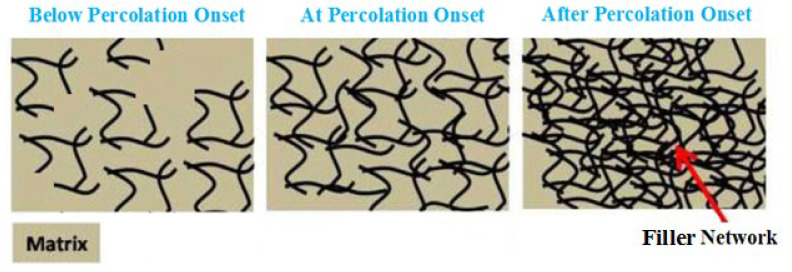
Schematic location of nanotubes in a nanocomposite before, during and after percolation onset.

The tough polymer–filler interfacial network, besides the large nanofiller superficial zone, often changes the polymer’s features due to adjacent nanoparticles leading to the formation of the interphase section [1,2,3,4,5,6,7,8,9,10,11,12,13,14,15]. This intermediate phase is different from both polymer media and nanoparticles. Several authors have tried to examine the interphase effects on the mechanics of nanocomposites [16,17,18]. The modeling approach in this field is highly challenging and interesting, since the analysis at the nanoscale in the interface region is a difficult task. Moreover, the interphase section around large and slender nanofiller such as halloysite nanotubes (HNTs) facilitates the development of networks within the sample, where the interphase eases the percolation onset and interlinks the nanoparticles before the physical contact [19,20,21,22]. However, the interphase section and its networking effectiveness for HNT-based systems were not argued in the previous articles. Due to the main reinforcing competences of the interphase section and filler network in nanocomposites, it is important to study them in HNT-filled systems. Figure 1 displays the location of nanotubes in a nanocomposite before, during and after percolation onset.

Recently, ceramic nanoparticles have attracted much interest in the academic research community [23,24,25,26,27,28,29,30,31,32]. Alumino-silicate and nanotubular HNT, for example, have the composition of Al_2_(OH)_4_Si_2_O_5_(2H_2_O) and few hydroxyl groups on their surface [30,33,34,35,36,37]. They normally have a single-tube construct, which can be dispersed in any solvent medium because of siloxane and their few hydroxyl groups, which simplifies the distribution and development of hydrogen bonding [38]. Moreover, HNTs are biocompatible, affordable, non-toxic and cheaper than other nanofillers such as carbon nanotubes (CNTs) [39,40]. These advantages indicate that HNTs are a good candidate for reinforcement in polymer media.

Many researchers have stated that the mixture of HNTs with polymer media enhances the thermal and mechanical properties [41,42,43,44]. Prashantha et al. [45] mixed neat HNT and quaternary ammonium salt-treated HNTs with polypropylene. They showed a fine dispersion of treated HNTs in comparison to the neat one, because of the tough interfacial network among polypropylene and treated HNTs. Krishnaiah et al. also functionalized the HNT with 3-aminopropyltriethoxysilane to improve the surface contact of the HNT with polypropylene and polylactide [46,47]. They also demonstrated that the fine dispersal of the treated HNT in the polymer matrix improved the mechanical and thermal performance of the composite.

Several authors have focused on the mechanical features of HNT-based systems through an experimental approach only [48,49,50]. Nevertheless, the theoretical models for this type of material and system are restricted due to insufficient scientific data and reports. Very few papers have considered the mechanical aspects of HNT nanocomposites, such as tensile strength and moduli [51,52]. Modified Halpin–Tsai and Pukanszky equations were utilized for calculating the tensile powers of a polylactide HNT system [51]. It was stated that an altered Halpin–Tsai equation overestimates the modulus, but the Pukanszky equation estimates acceptable results for strength. Additionally, it was suggested that a 3D finite element technique for polylactide HNT samples be included, hypothesizing an arbitrary HNT and assessing its predictive results [52]. Clearly, the modeling values for polymer HNT nanocomposites are highly insufficient, restricting the development of these materials and their applications. Therefore, novel theoretical models are an obligatory requirement for carrying out detailed scientific evaluation of the simulated interphase zone and networked HNTs.

Recently, our group published some papers on the modulus of polymer nanocomposites containing CNTs and HNTs [53,54]. We developed the Ouali model by considering the interphases around the dispersed and networked nanoparticles for the modulus of CNT-reinforced samples [53]. Moreover, we enhanced the advanced Takayanagi equation for calculating the modulus of HNT-based nanocomposites by considering the interphase around the dispersed and networked HNTs after percolation [54]. However, an Ouali model for the modulus of HNT-filled samples after percolation onset has not been developed yet. In this work, an Ouali model was developed for the calculation of the tensile modulus of polymer HNT systems after the percolation threshold by considering the function of networked HNTs and the surrounding interphase. The dispersed nanoparticles and surrounding interphase part were neglected, since they led to ineffective results for the system’s modulus. The formulated model includes the HNT size, interphase depth, percolation onset and HNT concentration as well as the volume portions and moduli of the networked HNTs and adjacent interphase. The components of the whole system’s modulus are explained to validate the formulated equation. Moreover, the tentative results of real samples are utilized to evaluate the model’s predictions. We believe that by employing this simple methodology in the current paper, we can develop a novel modeling method for the approximation of moduli in HNT-based systems.

## 2. Advanced Equations

The percolation onset of CNTs in the nanocomposites was connected to CNT size and interphase depth [55] by:

(1) $$\phi_{p}=\frac{\pi R^{2}l}{\frac{32}{3}\pi {\left(R+t\right)}^{3}[1+\frac{3}{4}(\frac{l}{R+t})+\frac{3}{32}{\left(\frac{l}{R+t}\right)}^{2}]}$$

where “*R*” and “*l*” demonstrate the radius and length of the HNT, individually. Additionally, “*t*” is interphase depth. This equation is usable for the HNT system, since both CNTs and HNTs have a similar tubular shape, and the percolation onset mainly correlates to filler shape and the surrounding interphase section. The interfacial adhesion by hydrogen, van der Waals or covalent bonds is considered by the interphase depth and modulus. Additionally, a thicker and stronger interphase presents stronger interfacial bonds within the samples.

The whole volume fraction of the interphase in the system comprising a tube-like filler such as an HNT is given [55] by:

(2) $$\phi_{i}=\phi_{f}[{\left(1+\frac{t}{R}\right)}^{2}-1]$$

where “$\phi_{f}$” is HNT volume fraction. This equation speculates the extent of the HNT and interphase besides the HNT amount, as expected.

The interphase area acts as an imperative, reinforcing efficiency in nanocomposites. Hence, both the HNT and nearby interphase toughen the specimens. The actual HNT amount equates the entire quantity of the reinforcing elements as:

(3) $$\phi_{eff}=\phi_{f}+\phi_{i}=\phi_{f}{\left(1+\frac{t}{R}\right)}^{2}$$

which regulates the reinforcing methods of the nanofiller and interphase by one factor.

Additionally, the proportion of a tubular nanofiller such as the HNT producing the network in the sample is expressed [56] by:

(4) $$f=\frac{\phi_{f}^{1/3}-\phi_{p}^{1/3}}{1-\phi_{p}^{1/3}}$$

The effective filler portion is further modified and given by the latter equation as:

(5) $$f=\frac{\phi_{eff}^{1/3}-\phi_{p}^{1/3}}{1-\phi_{p}^{1/3}}$$

Further, the original Ouali model and its developed form are expressed by the mentioned factors.

Ouali et al. [57] designed a model for the composite’s modulus by using the following percolation concept:

(6) $$E=\frac{(1-2\psi +\psi \phi_{f}^{})E_{m}^{}E_{f}^{}+(1-\phi_{f}^{})\psi E_{f}^{2}}{(1-\phi_{f}^{})E_{f}^{}+(\phi_{f}^{}-\psi )E_{m}^{}}$$

where “*E_m_*” is the medium’s modulus, and “*E_f_*” denotes the modulus of the filler. “ψ” correlates to percolation onset ($\phi_{p}^{}$), as given by:

(7) $$\begin{array}{c} \psi =0\\ \phi_{f}^{}\leq \phi_{p}^{} \end{array}$$

(8) $$\begin{array}{c} \psi =\phi_{f}^{}{\left(\frac{\phi_{f}^{}-\phi_{p}^{}}{1-\phi_{p}^{}}\right)}^{b}\\ \phi_{f}^{}>\phi_{p}^{} \end{array}$$

where “*b*” is an exponent, which obtains a value of 0.4 for a 3D structure [57].

When ψ=0, this model diminishes the converse roles of mixtures as:

(9) $$E=\frac{E_{m}^{}E_{f}^{}}{(1-\phi_{f}^{})E_{f}^{}+\phi_{f}^{}E_{m}^{}}$$

In our previous article [53], we developed the Ouali model using the isolated and networked nanofiller and neighboring interphase pieces after percolation onset. It was observed that the volume portions and moduli of the detached filler and adjoining interphase were ineffective on the modulus of nanocomposites [53]. Indeed, only the networks of particles and interphase controlled the stiffness of systems after percolation onset. Therefore, only the networked filler and surrounding interphase have the reinforcing components in the nanocomposites.

These terms extend the Ouali model to:

(10) $$E=\frac{(1-2\psi_{N}+\psi_{N}\phi_{N}^{})E_{m}^{}E_{N}^{}+(1-\phi_{N}^{})\psi_{N}E_{N}^{2}+(1-2\psi_{iN}+\psi_{iN}\phi_{iN}^{})E_{m}^{}E_{iN}^{}+(1-\phi_{iN}^{})\psi_{iN}E_{iN}^{2}}{(1-\phi_{N}^{})E_{N}^{}+(\phi_{N}^{}-\psi_{N})E_{m}^{}+(1-\phi_{iN}^{})E_{iN}^{}+(\phi_{iN}^{}-\psi_{iN})E_{m}^{}}$$

where “$\phi_{N}$” and “*E_N_*” are the volume portion and modulus of networked HNTs, in that order. Additionally, “$\phi_{iN}$” and “*E_iN_*” show the volume share and modulus of the interphase nearby the HNT network, correspondingly. Equation (10) considers the imperative components of the network and interphase.

Based on Equation (8), “$\psi_{N}$” and “$\psi_{iN}$”, after percolation starts, progress to:

(11) $$\psi_{N}=\phi_{f}^{}{\left(\frac{\phi_{f}^{}-\phi_{p}^{}}{1-\phi_{p}^{}}\right)}^{0.4}$$

(12) $$\psi_{iN}=\phi_{i}^{}{\left(\frac{\phi_{i}^{}-\phi_{p}^{}}{1-\phi_{p}^{}}\right)}^{0.4}$$

considering the roles of percolation onset and the amounts of both the HNT and the interphase.

Additionally, “$\phi_{N}$” and “$\phi_{iN}$” are calculated by:

(13) $$\phi_{N}=f\phi_{f}^{}$$

(14) $$\phi_{iN}=f\phi_{i}^{}$$

When “*f*” from Equation (5) is used as a replacement in the latter equations, “$\phi_{N}$” and “$\phi_{iN}$” are given by:

(15) $$\phi_{N}=\frac{\phi_{eff}^{1/3}-\phi_{p}^{1/3}}{1-\phi_{p}^{1/3}}\phi_{f}^{}$$

(16) $$\phi_{N}=\frac{\phi_{eff}^{1/3}-\phi_{p}^{1/3}}{1-\phi_{p}^{1/3}}\phi_{f}^{}\;.$$

assuming the roles of HNT size, interphase depth, HNT concnetration and percolation in the amounts of HNTs and interphase nets.

The relative modulus, *E*/*E_m_*, modifies the developed model (Equation (10)) to:

(17) $$E_{R}=\frac{(1-2\psi_{N}+\psi_{N}\phi_{N}^{})E_{N}^{}+(1-\phi_{N}^{})\psi_{N}E_{N}^{2}/E_{m}+(1-2\psi_{iN}+\psi_{iN}\phi_{iN}^{})E_{iN}^{}+(1-\phi_{iN}^{})\psi_{iN}E_{iN}^{2}/E_{m}}{(1-\phi_{N}^{})E_{N}^{}+(\phi_{N}^{}-\psi_{N})E_{m}^{}+(1-\phi_{iN}^{})E_{iN}^{}+(\phi_{iN}^{}-\psi_{iN})E_{m}^{}}$$

which undertakes the impacts of the networked HNT and neighboring interphase section on the improved stiffness of the HNT-based system. The above equations were developed for tubular nanofillers. In fact, the developed equations are only valid for the nanocomposites containing tubular nanoparticles.

## 3. Results and Discussion

The effects of all of the factors on the modulus of HNT-reinforced composites were elucidated to certify the formulated model.

**Figure 2:**
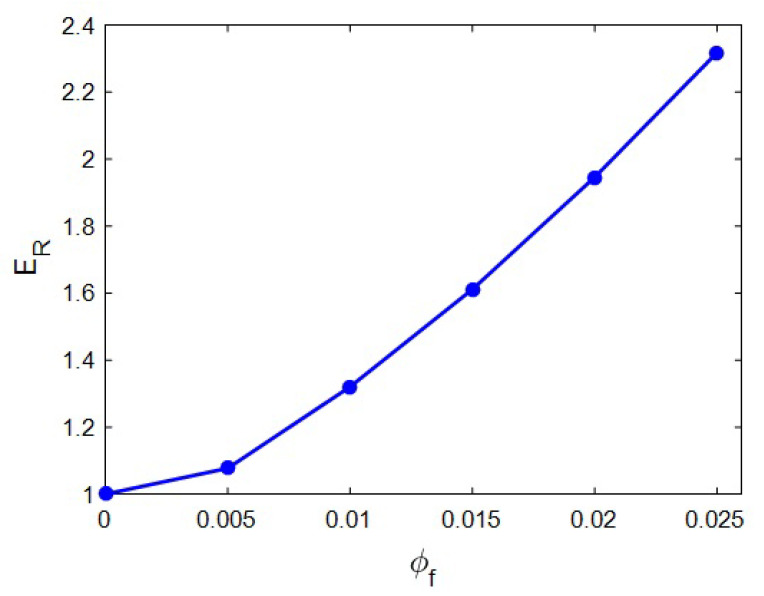
Predictions of “*E_R_*” at various HNT volume portions.

Figure 2 displays the “*E_R_*” estimated by the novel model at unlike values of HNT content and constant ranges of *E_m_* = 2 GPa, *R* = 20 nm, *l* = 2 μm, *t* = 20 nm, *E_N_* = 500 GPa and *E_iN_* = 100 GPa. After the addition of HNT content, the stiffness increased. “*E_R_*” reached 2.32 at the HNT volume share of 0.025. This level indicates that the HNT volume fraction of 0.025 increased the stiffness by 132%. However, the relative modulus had a low level at the minor ranges of HNT content.

This observation is reasonable, since the HNT was exceptionally firmer than the polymer matrix, and the adding of the HNT to the matrix enhanced the stiffness of samples. In fact, the extremely high difference between the moduli of the HNT and polymer caused the upgrading of the modulus in the samples, due to the better shifting of the load from polymer media to the stiff nanoparticles via the interphase section. All models for mechanical performance of the system demonstrated the same trend between the stiffness/strength of the system and filler concentration, since stiff particles commonly reinforce the system [58,59,60]. Accordingly, the advanced model logically estimated the effect of the HNT amount on the stiffness.

**Figure 3:**
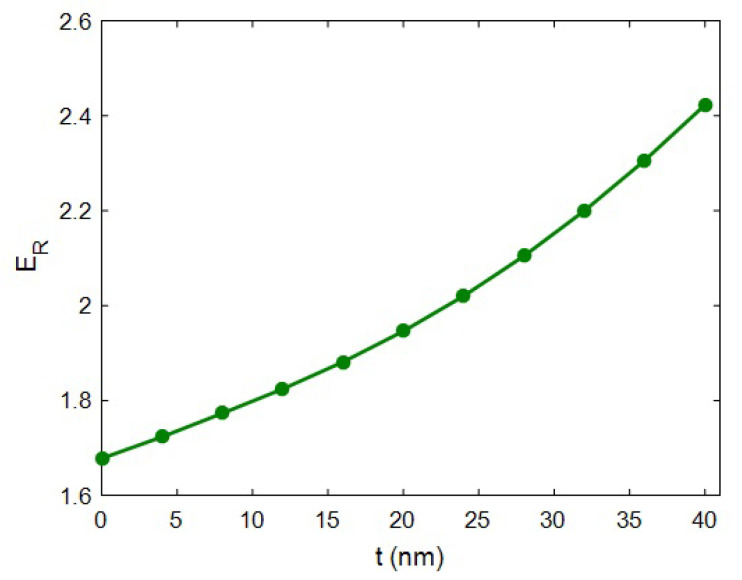
Relative modulus correlating to interphase depth (Equation (17)).

Figure 3 expresses the effect of interphase depth on the “*E_R_*” at *R* = 20 nm, $\phi_{f}$ = 0.02, *E_m_* = 2 GPa, *l* = 2 μm, *E_iN_* = 100 GPa and *E_N_* = 500 GPa. In the absence of the interphase area (*t* = 0), the relative modulus of 1.68 was calculated. Conversely, the relative modulus increased to 2.42 at *t* = 40 nm. As a result, the nanocomposite’s modulus significantly grew by thickening the interphase zone. This evidence indicates that the interphase depth is a vital factor in improving the stiffness of HNT-based systems. In fact, it is crucial to provide a dense interphase to enhance the modulus of HNT systems.

The interphase depth had positive effects on the effectiveness of HNT concentrations, percolation onset and the networking of the interphase (see Section 2). In fact, a thick interphase decreased the percolation onset and boosted the volume of the interphase zone in the system. During this stage, a larger interphase produced a more prominent network, which undoubtedly increased the stiffness of the system. In other words, a thicker interphase yielded an improved network in the system, thus increasing the resistance of the system against the load bearing. Instead, a narrow interphase created a small network, which undoubtedly diminished the rigidity of sample, owing to little stress bearing. This explanation validates the advanced equation for the modulus of this system.

**Figure 4:**
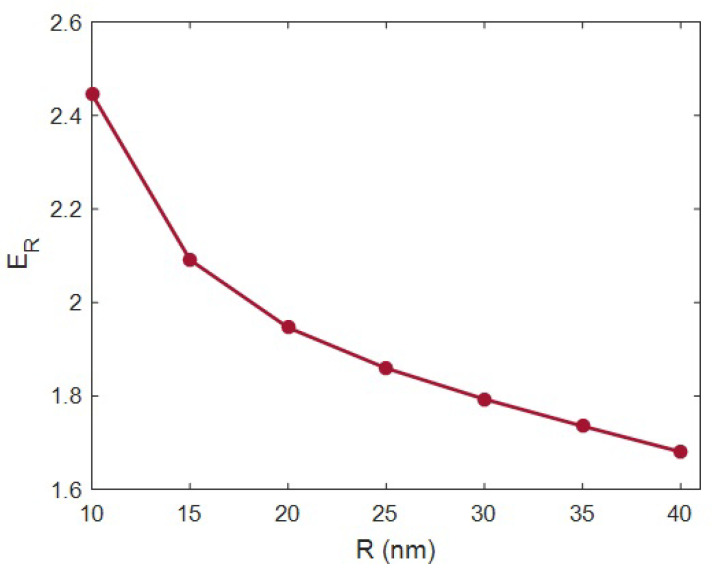
Influence of filler radius on the “*E_R_*”.

Figure 4 plots the estimations of “*E_R_*” at various orders of the HNT radius and *E_m_* = 2 GPa, $\phi_{f}$ = 0.02, *t* = 20 nm, *l* = 2 μm, *E_N_* = 500 GPa and *E_iN_* = 100 GPa according to the advanced model. An HNT radius of 10 nm caused *E_R_* = 2.45, while the “*E_R_*” was reduced to 1.68 at an HNT radius of 40 nm. Consequently, the HNT radius undesirably managed the stiffness, and the slender HNTs produced the stiffer samples. This observation is also crucial, since nanoparticles such as HNTs are inclined to aggregation/agglomeration during the fabrication of nanocomposites [61,62]. Indeed, it is vital to control the size of HNTs to obtain significant reinforcement within the sample.

The desirable role of thin HNTs in the modulus of samples is logical, because thin HNTs decrease the percolation onset and widen the interphase. According to the developed equations, thin HNTs produce a lower percolation onset and a highly effective filler fraction, which increases the network portion in the composites. Therefore, narrow HNTs certainly increase the modulus of samples, since they expand the interphase and the network stiffening within the samples. Conversely, thick HNTs cause slender interphase sections and few network interactions, which loosely reinforce the system. According to this explanation, the advanced model produced reasonable outputs at dissimilar levels of HNT radii.

**Figure 5:**
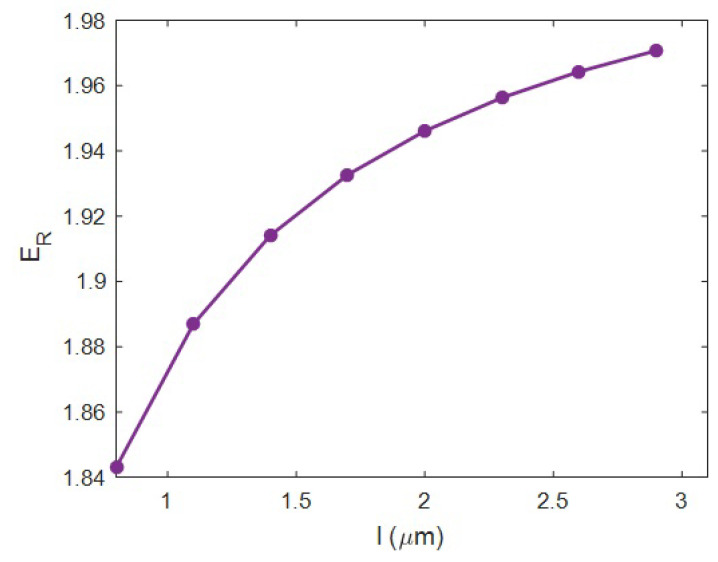
Dependency of relative modulus on the HNT length.

Figure 5 displays the discrepancy of “*E_R_*” at numerous intensities of HNT length and $\phi_{f}$ = 0.02, *E_m_* = 2 GPa, *t* = 20 nm, *R* = 20 nm, *E_iN_* = 100 GPa and *E_N_* = 500 GPa using the current model. E_R_ = 1.843 was observed at *l* = 0.8 μm, but “*E_R_*” increased to 1.972 at *l* = 3 μm. So, HNT size directly managed the stiffness, and large HNTs were needed to reinforce the samples. The large variation in HNT length from 0.8 to 3 μm only varied the relative modulus by about 0.13, which is a small amount.

HNT length influenced the percolation onset in the system. Large HNT shifted the percolation onset to a low filler concentration, which positively influenced the fraction of networked HNTs. So, it can be said that large HNTs produced a huge network within the system, whereas the short HNTs caused the small one. Since the extent of the network mainly controls the load bearing in the system, big HNTs advantageously influenced the stiffness of the system. Instead, short HNTs slightly manipulated the size of the network, yielding a minor reinforcing capacity. Generally, a higher aspect ratio of nanoparticles (length per diameter) obtains stiffer samples, due to the positive effects of large and narrow nanoparticles on the extents of the interphase section and networks [63,64] (see Section 2). Hence, the novel equation suitably predicted the role of HNT length in the rigidity of the system.

**Figure 6:**
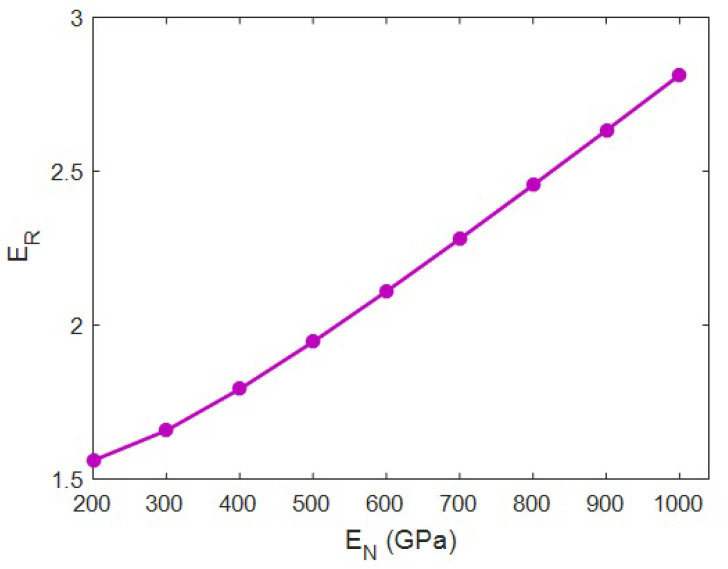
Relation between the moduli of HNT network and whole system according to Equation (17).

Figure 6 plots the effect of the net modulus on the “*E_R_*” at *E_m_* = 2 GPa, *R* = 20 nm, $\phi_{f}$ = 0.02, *l* = 2 μm, *t* = 20 nm and *E_iN_* = 100 GPa using the innovative model. The “*E_R_*” of 1.6 was developed at a net modulus of 200 GPa, but “*E_R_*” significantly grew to 2.8 at *E_N_* = 1000 GPa. These outputs display the direct correlation between the moduli of system and its network. A stronger network produced a stiffer sample, whereas poor networks ineffectively reinforced the sample. The big range of the relative modulus from 1.6 to 2.8 verifies that the net modulus is important for the reinforcement of the system. Consequently, researchers should try to extend the HNT network in the system to achieve a better stiffening efficiency.

The stiffer network in the nanocomposite bore higher amounts of stress. However, the poorer network could not stand against the stress and breaks during loading. Therefore, the stiffer network induced the higher modulus in the system, but the poorer network contrarily weakened the sample. In fact, there was a direct link between the moduli of the network and the whole system; meanwhile, the stiffness of the network mainly manipulated the stiffness of the whole system. This explanation confirms the approximations of the model at various ranks of net modulus. This relation was also reported in the earlier research on the stiffness/strength of samples [53,65].

**Figure 7:**
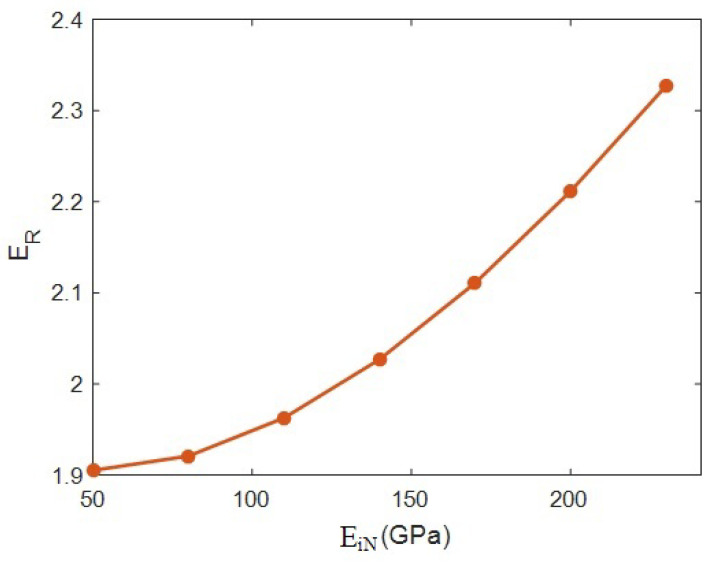
Outputs of Equation (17) at numerous values of interphase net modulus.

Figure 7 expresses the model’s estimations correlating to the interphase modulus at *R* = 20 nm, $\phi_{f}$ = 0.02, *E_m_* = 2 GPa, *t* = 20 nm, *l* = 2 μm and *E_N_* = 500 GPa. The relative modulus was 1.9 at *E_iN_* = 50 GPa; nonetheless, the “*E_R_*” increased to 2.33 at *E_iN_* = 250 GPa. These calculations establish that the stiffness grew by 43% when the modulus of the interphase net varied from 50 to 250 GPa. As a result, the stiffness of the networked interphase directly influenced the overall stiffness. This output indicates that the modulus of the interphase network is a main factor influencing the modulus of the whole sample.

The modulus of the networked interphase is also imperative, since the interphase section around the network is a bridge transferring the stress from poor polymer media to the HNT network. A firmer interphase zone neighboring the network undoubtedly transferred a higher stress amount, but a defective interphase declined during stress charging. Hence, a tough interphase network positively grew the modulus of the whole system, since it can tolerate further stress. In contrast, a poorer interphase network accepted a lower volume of stress, which is inefficient for the stiffness of the whole system. Therefore, the innovative model suitably predicted the effects of the interphase net modulus on the stiffness of the system.

**Figure 8:**
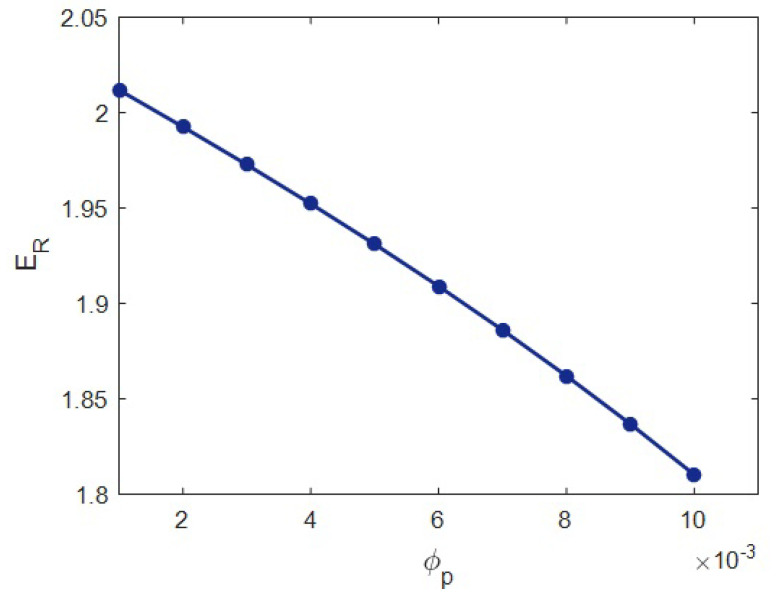
The calculations of formulated model at several series of percolation onset.

Figure 8 projects the results of the present model at several values of percolation onset at constant *R* = 20 nm, *E_m_* = 2 GPa, $\phi_{f}$ = 0.02, *t* = 20 nm, *l* = 2 μm, *E_N_* = 500 GPa and *E_iN_* = 100 GPa. “*E_R_*” was 2.01 at the percolation onset of 0.001, but the relative modulus declined to 1.81 when the percolation onset increased to 0.01. Therefore, a higher modulus was observed at a lower percolation onset, and it is essential to decrease the percolation onset, leading to stiffness. Indeed, the percolation onset adversely controlled the modulus of samples (see Section 2). This occurrence is expected, since a low percolation beginning boosts the networked HNT component (Equation (5)). A lower percolation onset increases the network size, which increases the modulus. Contrariwise, a high percolation induces the smaller networks of HNT and interphase, due to the low value of “*f*” (see Equation (5)) in this condition. Consequently, the percolation onset unfavorably governs the stiffness, due to its effect on the extent of network. This description validates the results of Equation (17) at several ranges of percolation onset.

**Figure 9:**
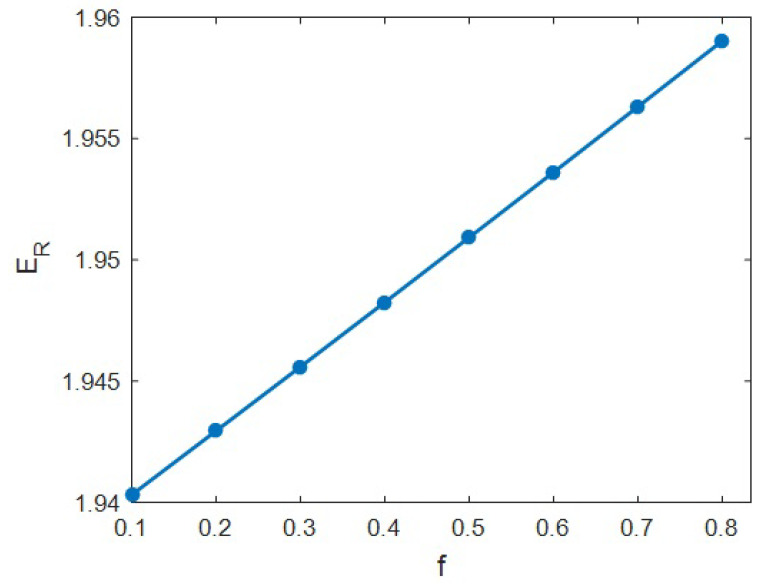
A plot between “*E_R_*” and the portion of network using the advanced model.

Figure 9 establishes the role of “*f*” as the portion of the filler–interphase network in the modulus of the system. *f* = 0.1 produced *E_R_* = 1.94; nonetheless, the “*E_R_*” rose to 1.958 at *f* = 0.8. These results signify the direct connection between the stiffness and the share of networked HNTs or interphase. However, numerous points of “f” from 0.1 to 0.8 slightly changed the relative modulus.

“*f*” determines the size of networks (both interphase and HNT) in the system. A large “*f*” depicts the contribution of numerous HNTs and a large interphase to the network (Equations (15) and (16)). So, a larger “*f*” shows the creation of larger networks of HNT and interphase within the system, which certainly raises the modulus. Conversely, a lower “*f*” reveals the involvement of few HNTs or decreased interphase section in the network, which is undesirable for the stiffness of system. In summary, “*f*” reveals the size of networked interphase and HNTs, and thus “*f*” directly controls the stiffness of samples, due to the effective role of network size in the load bearing. Consequently, the novel model appropriately foresaw the impact of “*f*” on the stiffness of HNT-based samples.

**Figure 10:**
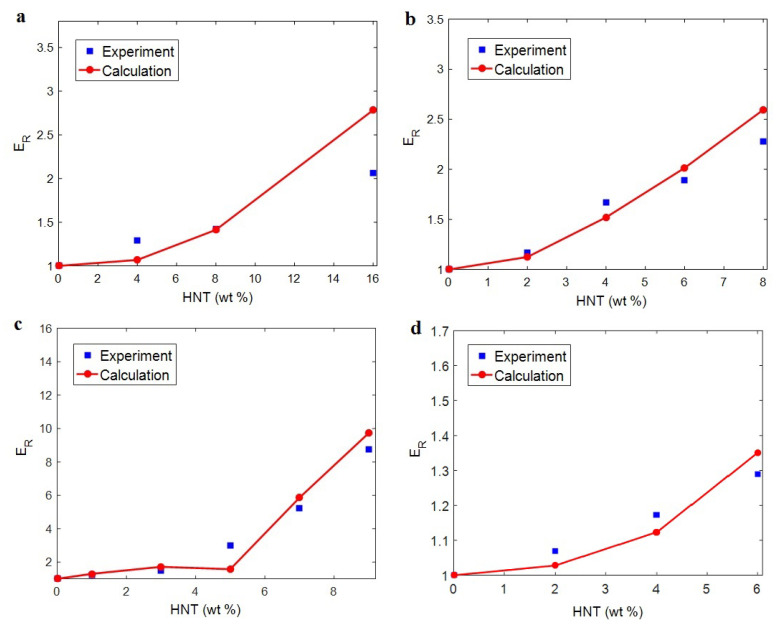
Experimental “*E_R_*” and the calculations of established technique for (**a**) PA12 [66], (**b**) cellulose [67], (**c**) starch [68] and (**d**) PLA [51] systems containing HNT.

Now, the outputs of Equation (17) are correlated to the tentative numbers of the modulus for real examples. Table 1 depicts HNT systems and their details (*E_m_*, *R* and *l*) from valid published articles. Further details about this system are accessible in the original references. These data are substituted into the developed equations and the final model (Equation (17)) to analyze the “*E_R_*” for the required study specimens. Figure 10 displays the measured and hypothetical extents of “*E_R_*” for the given examples. It is depicted that all theoretical values matched the measured numbers at entire HNT weight fractions. Hence, the innovative model successfully predicted the modulus of real samples, and thus the developed model offers a valid equation for the modulus of HNT-filled systems when compared to experimental values.

The values of the interphase and network properties were also calculated, as listed in Table 1. “*t*”, as interphase depth, changed from 3 to 15 nm for the current samples. These results are significant, since they were obtained at the nanoscale. The thickest and the thinnest interphases were detected for polylactide (PLA) and polyamide 12 (PA12) samples, respectively. When HNT size and interphase depth were replaced in Equation (1), the percolation onset was estimated. The lowest and the highest percolation were found for cellulose and PA12 samples, correspondingly. These outputs indicate that the network in cellulose samples is established at an extremely low HNT content. However, the network in PA12 needs more HNTs (high HNT concentration) to be constructed, due to the low level of interphase depth.

The modulus of the interphase network was estimated by the predictions of the current model. When the experimented moduli of nanocomposites were compared to the developed model, numerous values for “*E_iN_*” were obtained, and the average interphase modulus was reported for each sample. The values of the interphase net modulus were also shown to range from 20 to 45 GPa. The strongest and the weakest interphase networks were found in PLA and PA12 samples, respectively. Furthermore, the modulus of the HNT network in the samples was obtained and found to range from 150 to 250 GPa. The strongest network was observed in the cellulose system, but PA12 and starch examples show the same network modulus for 150 GPa. The firm and vast networks of the interphase section and HNT led to more significant improvements in the modulus of the samples, thus equally validating the existing data obtained with the newly modified equations.

## 4. Conclusions

The reported and earlier published literature studies have ignored the interphase–HNT networks in the HNT-reinforced systems. Moreover, they used complex and undefined equations, which baffled readers. In this work, the Ouali model was developed for a polymer HNT system assuming the networks of the interphase and nearby HNT after percolation. The developed model in the current study considers the properties of interphase–HNT networks after percolation onset through simple, meaningful equations and parameters, which is the prime novelty and advantage of this study. It is highly evident from the data that the HNT content directly influenced the system modulus, and “*E_R_*” reached 2.32 at an HNT volume share of 0.025. *t* = 0 led to the “*E_R_*” of 1.68, although the “*E_R_*” increased to 2.42 at *t* = 40 nm. So, a stronger interphase is vital to obtaining a better modulus. An HNT radius of 10 nm produced *E_R_* = 2.45, while the “*E_R_*” was reduced to 1.68 at an HNT radius of 40 nm. Thus, narrow HNTs are desirable for the system. HNT length directly controlled the stiffness, but it had a non-significant effect. The “*E_R_*” was 1.6 at a net modulus of 200 GPa; however, the “*E_R_*” considerably improved to 2.8 at *E_N_* = 1000 GPa. Additionally, *E_R_* = 1.9 at *E_iN_* = 50 GPa, but the “*E_R_*” grew to 2.33 at *E_iN_* = 250 GPa. Consequently, the stronger networks of HNTs or surrounding interphase section produced a stiffer sample. A higher “*E_R_*” was observed at a lower percolation onset, and it is important to decrease the percolation onset to reinforce the system. There was a direct connection between the stiffness and the portion of networks. The predicted calculations of Equation (17) acceptably match the experimental data of many recorded systems. This is another indication for the significant validation of Equation (17). The highest HNT content, the strongest interface between the polymer and HNT (the thickest interphase), the thinnest and the longest HNT and the strongest networks of HNT and interphase produced the lowest percolation onset and the biggest/strongest networks providing the toughest samples.

## Figures and Tables

**Table 1:** Properties for HNT examples and the estimations of developed equations.

No.	Samples [Ref.]	*E_m_* (GPa)	*R* (nm)	*l* (μm)	*t* (nm)	$\phi_{p}$	*E_iN_* (GPa)	*E_N_* (GPa)
1	PA12/HNT [66]	1.55	35	1.0	3.0	0.024	20	150
2	Cellulose/HNT [67]	1.80	25	2.0	6.0	0.009	40	250
3	Starch/HNT [68]	0.08	35	1.2	9.0	0.018	25	150
4	PLA/HNT [51]	2.90	40	1.2	15	0.018	45	220

## Data Availability

The datasets used and/or analyzed during the current study are available from the corresponding author on reasonable request.

## References

[1] Dassan E.G.B., Ab Rahman A.A., Abidin M.S.Z., Akil H. (2020). Carbon nanotube–reinforced polymer composite for electromagnetic interference application: A review. Nanotechnol. Rev..

[2] Li J., Zhang Z., Fu J., Liang Z., Ramakrishnan K.R. (2021). Mechanical properties and structural health monitoring performance of carbon nanotube-modified FRP composites: A review. Nanotechnol. Rev..

[3] Hassanzadeh-Aghdam M.K., Mahmoodi M.J., Ansari R. (2018). Creep performance of CNT polymer nanocomposites–An emphasis on viscoelastic interphase and CNT agglomeration. Compos. Part B Eng..

[4] Zare Y., Rhee K.Y., Park S.-J. (2018). A modeling methodology to investigate the effect of interfacial adhesion on the yield strength of MMT reinforced nanocomposites. J. Ind. Eng. Chem..

[5] Zare Y., Daraei A., Vatani M., Aghasafari P. (2013). An analysis of interfacial adhesion in nanocomposites from recycled polymers. Comput. Mater. Sci..

[6] Zare Y., Rhee K.Y. (2017). Development of Hashin-Shtrikman model to determine the roles and properties of interphases in clay/CaCO3/PP ternary nanocomposite. Appl. Clay Sci..

[7] Zare Y., Rhee K.Y. (2020). A simple and sensible equation for interphase potency in carbon nanotubes (CNT) reinforced nanocomposites. J. Mater. Res. Technol..

[8] Kazemi F., Mohammadpour Z., Naghib S.M., Zare Y., Rhee K.Y. (2021). Percolation onset and electrical conductivity for a multiphase system containing carbon nanotubes and nanoclay. J. Mater. Res. Technol..

[9] Wan C., Chen B. (2012). Reinforcement and interphase of polymer/graphene oxide nanocomposites. J. Mater. Chem..

[10] Zare Y. (2016). Modeling approach for tensile strength of interphase layers in polymer nanocomposites. J. Colloid Interface Sci..

[11] Zare Y. (2016). Study on interfacial properties in polymer blend ternary nanocomposites: Role of nanofiller content. Comput. Mater. Sci..

[12] Zare Y., Rhee K.Y. (2019). Significances of interphase conductivity and tunneling resistance on the conductivity of carbon nanotubes nanocomposites. Polym. Compos..

[13] Zare Y., Rhee K.Y. (2020). Analysis of critical interfacial shear strength between polymer matrix and carbon nanotubes and its impact on the tensile strength of nanocomposites. J. Mater. Res. Technol..

[14] Bhat A., Budholiya S., Raj S.A., Sultan M.T.H., Hui D., Shah A.U., Safri S.N.A. (2021). Review on nanocomposites based on aerospace applications. Nanotechnol. Rev..

[15] Jian W., Hui D., Lau D. (2020). Nanoengineering in biomedicine: Current development and future perspectives. Nanotechnol. Rev..

[16] Tharu S.A., Panchal M.B. (2020). Effect of interphase on elastic and shear moduli of metal matrix nanocomposites. Eur. Phys. J. Plus.

[17] Yang Y., He Q., Rao Y., Dai H. (2019). Estimation of dynamic thermo viscoelastic moduli of short fiber-reinforced polymers based on a micromechanical model considering interphases/interfaces conditions. Polym. Compos..

[18] Zamanian M., Ghasemi F.A., Mortezaei M. (2020). Interphase characterization and modeling of tensile modulus in epoxy/silica nanocomposites. J. Appl. Polym. Sci..

[19] Zare Y., Rhee K.Y. (2019). Tensile modulus prediction of carbon nanotubes-reinforced nanocomposites by a combined model for dispersion and networking of nanoparticles. J. Mater. Res. Technol..

[20] Zare Y., Rhee K.Y. (2020). Development of Conventional Paul Model for Tensile Modulus of Polymer Carbon Nanotube Nanocomposites After Percolation Threshold by Filler Network Density. JOM.

[21] Zare Y., Rhim S., Garmabi H., Rhee K.Y. (2018). A simple model for constant storage modulus of poly (lactic acid)/poly (ethylene oxide)/carbon nanotubes nanocomposites at low frequencies assuming the properties of interphase regions and networks. J. Mech. Behav. Biomed. Mater..

[22] Zare Y., Rhee K.Y. (2020). Simulation of Young’s modulus for clay-reinforced nanocomposites assuming mechanical percolation, clay-interphase networks and interfacial linkage. J. Mater. Res. Technol..

[23] Abdollahi Boraei S.B., Esmaeili Bidhendib M., Afzali D. (2017). Preparation of SiO_2_/ZrO_2_ ceramic nanocomposite coating on Aluminum alloys as metallic part of the photovoltaic cells and study its corrosion behavior. Environ. Energy Econ. Res..

[24] Abdollahi B., Afzali D., Hassani Z. (2018). Corrosion inhibition properties of SiO_2_-ZrO_2_ nanocomposite coating on carbon steel 178. Anti-Corros. Methods Mater..

[25] Abdollahi Boraei S.B., Esmaeili-Bidhendi M., Afzali D., Hashemi R. (2018). Preparation of SiO_2_/_TiO2_ ceramic nano composite coating by sol-gel method on carbon steel and study the properties of it against corrosive ion in treated wastewater. J. Sci. Technol. Compos..

[26] Moradi S., Yeganeh J.K. (2020). Highly toughened poly(lactic acid) (PLA) prepared through melt blending with ethylene-co-vinyl acetate (EVA) copolymer and simultaneous addition of hydrophilic silica nanoparticles and block copolymer compatibilizer. Polym. Test..

[27] Yeganeh J.K. (2017). Dynamics of nucleation and growth mechanism in the presence of nanoparticles or block copolymers: Polystyrene/poly(vinyl methyl ether). Polym. Bull..

[28] Bakhtiari A., Ghasemi F.A., Naderi G., Nakhaei M.R. (2020). An approach to the optimization of mechanical properties of polypropylene/nitrile butadiene rubber/halloysite nanotube/polypropylene-*g*-maleic anhydride nanocomposites using response surface methodology. Polym. Compos..

[29] Pourmohammadi-Mahunaki M., Haddadi-Asl V., Roghani-Mamaqani H., Koosha M., Yazdi M. (2020). Halloysite-reinforced thermoplastic polyurethane nanocomposites: Physico-mechanical, rheological, and thermal investigations. Polym. Compos..

[30] Cheng C., Song W., Zhao Q., Zhang H. (2020). Halloysite nanotubes in polymer science: Purification, characterization, modification and applications. Nanotechnol. Rev..

[31] Zhang M., Wang L., Yan H., Lian L., Si J., Long Z., Cui X., Wang J., Zhao L., Yang C. (2021). Palladium-halloysite nanocomposites as an efficient heterogeneous catalyst for acetylene hydrochlorination. J. Mater. Res. Technol..

[32] Al Rashid A., Khan S.A., Al-Ghamdi S.G., Koç M. (2021). Additive manufacturing of polymer nanocomposites: Needs and challenges in materials, processes, and applications. J. Mater. Res. Technol..

[33] Yang T., Lu S., Song D., Zhu X., Almira I., Liu J., Zhu Y. (2021). Effect of Nanofiller on the Mechanical Properties of Carbon Fiber/Epoxy Composites under Different Aging Conditions. Materials.

[34] Lampropoulou P., Papoulis D. (2021). Halloysite in Different Ceramic Products: A Review. Materials.

[35] Stavitskaya A., Fakhrullina G., Nigamatzyanova L., Sitmukhanova E., Khusnetdenova E., Fakhrullin R., Vinokurov V. (2021). Biodistribution of Quantum Dots-Labelled Halloysite Nanotubes: A *Caenorhabditis elegans* In Vivo Study. Materials.

[36] Haroosh H.J., Dong Y., Jasim S., Ramakrishna S. (2021). Improvement of Drug Release and Compatibility between Hydrophilic Drugs and Hydrophobic Nanofibrous Composites. Materials.

[37] Boraei S.B.A., Nourmohammadi J., Mahdavi F.S., Zare Y., Rhee K.Y., Montero A.F., Herencia A.J.S., Ferrari B. (2022). Osteogenesis capability of three-dimensionally printed poly(lactic acid)-halloysite nanotube scaffolds containing strontium ranelate. Nanotechnol. Rev..

[38] Du M., Guo B., Lei Y., Liu M., Jia D. (2008). Carboxylated butadiene–styrene rubber/halloysite nanotube nanocomposites: Interfacial interaction and performance. Polymer.

[39] Duong H.M., Tran T.Q., Kopp R., Myint S.M., Peng L. (2019). Direct Spinning of Horizontally Aligned Carbon Nanotube Fibers and Films from the Floating Catalyst Method. Nanotube Superfiber Materials.

[40] Lepak-Kuc S., Taborowska P., Tran T., Duong H., Gizewski T., Jakubowska M., Patmore J., Lekawa-Raus A. (2020). Washable, colored and textured, carbon nanotube textile yarns. Carbon.

[41] Pourmohammadi-Mahunaki M., Haddadi-Asl V., Roghani-Mamaqani H., Koosha M., Yazdi M. (2020). Preparation of polyurethane composites reinforced with halloysite and carbon nanotubes. Polym. Compos..

[42] Li R., Zhang Y., Lin Z., Lei Q., Liu Y., Li X., Liu M., Wu G., Luo S., Wang H. (2021). Injectable halloysite-g-chitosan hydrogels as drug carriers to inhibit breast cancer recurrence. Compos. Part B Eng..

[43] Afshoun H.R., Pourafshari Chenar M., Moradi M.R., Ismail A.F., Matsuura T. (2020). Effects of halloysite nanotubes on the morphology and CO_2_/CH_4_ separation performance of Pebax/polyetherimide thin-film composite membranes. J. Appl. Polym. Sci..

[44] Aguiar R., Miller R., Petel O.E. (2020). Synthesis and characterization of partially silane-terminated polyurethanes reinforced with acid-treated halloysite nanotubes for transparent armour systems. Sci. Rep..

[45] Prashantha K., Lacrampe M.F., Krawczak P. (2011). Processing and characterization of halloysite nanotubes filled polypropylene nanocomposites based on a masterbatch route: Effect of halloysites treatment on structural and mechanical properties. Express Polym. Lett..

[46] Krishnaiah P., Ratnam C.T., Manickam S. (2017). Development of silane grafted halloysite nanotube reinforced polylactide nanocomposites for the enhancement of mechanical, thermal and dynamic-mechanical properties. Appl. Clay Sci..

[47] Krishnaiah P., Manickam S., Ratnam C.T., Raghu M., Parashuram L., Kumar S.P., Jeon B.-H. (2020). Mechanical, thermal and dynamic-mechanical studies of functionalized halloysite nanotubes reinforced polypropylene composites. Polym. Polym. Compos..

[48] Gaaz T.S., Luaibi H., Al-Amiery A.A., Kadhum A.A.H. (2018). Effect of phosphoric acid on the morphology and tensile properties of halloysite-polyurethane composites. Results Phys..

[49] Bidsorkhi H.C., Adelnia H., Pour R.H., Soheilmoghaddam M. (2015). Preparation and characterization of ethylene-vinyl acetate/halloysite nanotube nanocomposites. J. Mater. Sci..

[50] Govindasamy K., Dahlan N.A., Janarthanan P., Goh K.L., Chai S.-P., Pasbakhsh P. (2020). Electrospun chitosan/polyethylene-oxide (PEO)/halloysites (HAL) membranes for bone regeneration applications. Appl. Clay Sci..

[51] Prashantha K., Lecouvet B., Sclavons M., Lacrampe M.F., Krawczak P. (2013). Poly (lactic acid)/halloysite nanotubes nanocomposites: Structure, thermal, and mechanical properties as a function of halloysite treatment. J. Appl. Polym. Sci..

[52] De Silva R.T., Pasbakhsh P., Goh K.-L., Mishnaevsky L. (2014). 3-D computational model of poly (lactic acid)/halloysite nanocomposites: Predicting elastic properties and stress analysis. Polymer.

[53] Zare Y., Rhee K.Y. (2017). Development and modification of conventional Ouali model for tensile modulus of polymer/carbon nanotubes nanocomposites assuming the roles of dispersed and networked nanoparticles and surrounding interphases. J. Colloid Interface Sci..

[54] Zare Y., Rhee K.Y. (2022). Development of a model for modulus of polymer halloysite nanotube nanocomposites by the interphase zones around dispersed and networked nanotubes. Sci. Rep..

[55] Zare Y., Rhee K.Y., Park S.-J. (2019). Modeling the roles of carbon nanotubes and interphase dimensions in the conductivity of nanocomposites. Results Phys..

[56] Feng C., Jiang L. (2013). Micromechanics modeling of the electrical conductivity of carbon nanotube (CNT)–polymer nanocomposites. Compos. Part A Appl. Sci. Manuf..

[57] Ouali N., Cavaillé J., Perez J. (1991). Elastic, viscoelastic and plastic behavior of multiphase polymer blends. Plast. Rubber Compos. Processing Appl..

[58] Zare Y., Rhee K.Y. (2021). The strengthening efficacy of filler/interphase network in polymer halloysite nanotubes system after mechanical percolation. J. Mater. Res. Technol..

[59] Zare Y., Rhee K.Y., Park S. (2021). Tensile strength of carbon-nanotube-based nanocomposites by the effective characteristics of interphase area nearby the filler network. Polym. Compos..

[60] Zare Y., Rhee K.Y. (2020). Modeling the Effects of Filler Network and Interfacial Shear Strength on the Mechanical Properties of Carbon Nanotube-Reinforced Nanocomposites. JOM.

[61] Tan H., Gu B., Guo Y., Ma B., Huang J., Ren J., Zou F., Guo Y. (2018). Improvement in compatibility of polycarboxylate superplasticizer with poor-quality aggregate containing montmorillonite by incorporating polymeric ferric sulfate. Constr. Build. Mater..

[62] Shokri-Oojghaz R., Moradi-Dastjerdi R., Mohammadi H., Behdinan K. (2018). Stress distributions in nanocomposite sandwich cylinders reinforced by aggregated carbon nanotube. Polym. Compos..

[63] Chen Y., Pan F., Guo Z., Liu B., Zhang J. (2015). Stiffness threshold of randomly distributed carbon nanotube networks. J. Mech. Phys. Solids.

[64] Hao B., Mu L., Ma Q., Yang S., Ma P.-C. (2018). Stretchable and compressible strain sensor based on carbon nanotube foam/polymer nanocomposites with three-dimensional networks. Compos. Sci. Technol..

[65] Huang T.-M., Lin C.-K., Wu R.-J., Liu Y.-T., Hsieh W.-Y., Chang J.-H. (2019). Development of segregated 3D graphene networks in rubber nanocomposites with enhanced electrical and mechanical properties. J. Polym. Res..

[66] Lecouvet B., Sclavons M., Bourbigot S., Bailly C. (2014). Towards scalable production of polyamide 12/halloysite nanocomposites via water-assisted extrusion: Mechanical modeling, thermal and fire properties. Polym. Adv. Technol..

[67] Soheilmoghaddam M., Wahit M.U. (2013). Development of regenerated cellulose/halloysite nanotube bionanocomposite films with ionic liquid. Int. J. Biol. Macromol..

[68] He Y., Kong W., Wang W., Liu T., Liu Y., Gong Q., Gao J. (2012). Modified natural halloysite/potato starch composite films. Carbohydr. Polym..

